# A Novel Dual-Band Six-Phase Voltage-Control Oscillator †

**DOI:** 10.3390/s18114025

**Published:** 2018-11-19

**Authors:** San-Fu Wang, Yu-Wei Chang, Chun-Yen Tang

**Affiliations:** 1 Department of Electronic Engineering, National Chin-Yi University of Technology, Taiping, Taichung 41170, Taiwan; 2 Department of Electronic Engineering, Ming Chi University of Technology, New Taipei City 24301, Taiwan; M05158008@mail2.mcut.edu.tw (Y.-W.C.); tata0101@yahoo.com.tw (C.-Y.T.)

**Keywords:** delay cell, jitter, ring oscillator, voltage control oscillator

## Abstract

The paper presents a novel dual-band six-phase voltage-control oscillator. The voltage-controlled oscillator (VCO) with a single-ended delay cell architecture has a lower power consumption, a smaller chip area, and a larger output swing than one with a differential delay cell architecture. However, the conventional even-phase outputs ring-type VCO cannot be implemented using single-ended delay cells. In other words, the VCO with single-ended delay cells meets most of the requirements of a sensor circuit system, except even-phase outputs function. This work presents a dual-band six-phase ring type VCO, which is implemented using the proposed single-ended delay cell. The proposed VCO both exhibits the advantages of single-ended delay cells and differential delay cells. The proposed delay cell has a band-switching function, which improves the jitter performance of a VCO in which it is used. The proposed VCO can be operated at 890–1080 MHz. The peak-to-peak jitter and the root mean square jitter are the 35.5 ps and 2.8 ps (at 1 GHz), respectively. The maximal power consumption is approximately 6.4 mW at a supply voltage of 1.8 V in a United Microelectronics Corporation 0.18 μm RF CMOS process. The area of the chip is 0.195 × 0.208 mm^2^.

## 1. Introduction

Over the last few years, optical sensor devices, medical sensor devices, and radar sensor devices have been extensively used in Internet of Things (IoT) systems. These sensor devices help the IoT systems collect large amounts of data. Therefore, these sensor devices are the basic devices used in big data research [1,2,3,4,5]. However, these devices require low power circuits to extend device life time. Small size and low cost make the device more popular, and good circuit characteristics to improve the performance of the device. For example, a low power low phase noise differential ring oscillator can effectively increase the use time of the Medical Implantable Communications Service (MICS) transceivers [3]. The integration of analog-to-digital converter, voltage-controlled oscillator, receiver, transmitter and digital circuits on a single chip reduces the size and cost of the sensor system [4]. A low-jitter and low-reference-spur ring-type voltage-controlled oscillator provides a purer clock signal for digital circuits, and effectively reduces the erroneous operation of the digital circuit [6]. A 1–9 GHz linear-wide-tuning-range quadrature ring oscillator uses Doppler radar sensor to increase frequency range and accuracy [7]. A single-ended delay cell based VCO with favorable power consumption and supply voltage can be implemented without a tail current circuit, but it has the disadvantages of low operating frequency and poor jitter performance [8]. A new differential delay cell based VCO has good phase noise performance, but its power consumption is too large, which is unsuitable for using in sensor systems [9]. A new differential delay cell based VCO provides high operating frequency, but it also consumes too much power and is not suitable for using in a sensor system [10]. Based on the above reasons, voltage-control oscillators (VCOs) are important components of sensor device.

**Figure 1:**
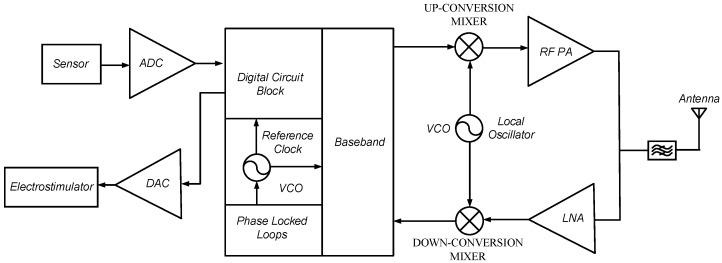
The block diagram of Medical Implantable Communications Service (MICS) transceivers.

Figure 1 shows the block diagram of Medical Implantable Communications Service (MICS) transceivers. Obviously, whether it is the reference clock circuit [6,11] or local oscillator circuit [3,4,12], the VCO is an indispensable component in sensor devices. Therefore, the characteristics of the VCO affect the performance of the sensor products [6,11,13].

Most of the reference clock circuit and part of the local oscillator circuit are implemented with a ring structure [1,13,14]. These VCOs are implemented by single-ended delay cells or differential delay cells [1,3]. A single-ended delay cell provides a larger output swing, a smaller chip area, a lower dissipated current, and a less complex design than a differential delay cell [3,8,15,16]. However, VCOs that are based on single-ended delay cells cannot provide even-phase outputs. VCOs with symmetrical even-phase outputs are more widely used than VCOs with odd-phase outputs, because a VCO with symmetrical even-phase outputs can satisfy more requirements of electronic circuits, such as symmetry trigger circuits, sample and hold circuits, charge pump circuits, and others, in sensor devices. All of these circuits require symmetrical even phase clock signals as trigger signals or control signals. A conventional VCO with odd-phase outputs does not meet this requirement. Consequently, an even-phase output ring VCO that uses a single-ended delay cell has significant needs.

The proposed novel dual-band symmetrical six-phase voltage-control oscillator consists of two single-ended delay-cell odd-phase ring oscillators. The proposed VCO architecture can generate outputs of odd-phase and even-phase, where the odd-phase is 3-phase, 5-phase, 7-phase, …, (2n + 1)-phase and the even-phase is 6-phase, 10-phase, 14-phase, …, 2(2n + 1)-phase. In addition, the proposed VCO architecture has the advantages of the single-ended delay cells and the functions of differential delay cells. Hence, it can satisfy different sensors and different applications in the IoT systems. In this research, the six-phase outputs and 1 GHz operation frequency are used to verify the functionality and performance of the proposed VCO and single-ended delay cell.

## 2. Proposed Circuit

The conventional single-ended ring-type oscillator consists of a series of inverting amplifiers in a feedback loop, and each stage of an inverting amplifier provides a phase shift of 180 degrees. To satisfy the Barkhausen criterion, ∠H(jω0) = 180° [17], a ring-type oscillator can only consist of an odd number of single-ended delay cells. Therefore, the conventional ring-type oscillator that uses single-ended delay cells cannot provide symmetrical even-phase outputs [18,19,20,21].

Conventional symmetrical even-phase output VCOs are typically constituted using differential delay cells, whose implementation requires tail current circuits [9,10]. Therefore, the output swing of such a VCO is limited by the voltage of the tail current circuit. The operating frequency of the VCO is adjusted by controlling the current through the differential delay cell. Therefore, the power consumption and output swing of the VCO vary considerably with operational frequency, greatly increasing the complexity of the design of the sensor circuit system.

**Figure 2:**
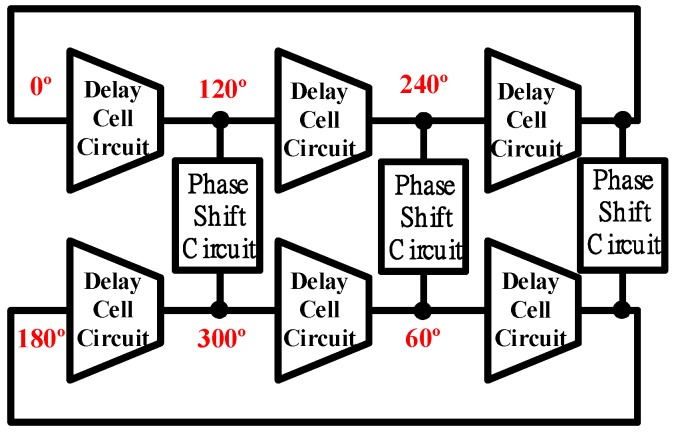
Proposed dual-band six-phase voltage-control oscillator.

**Figure 3:**
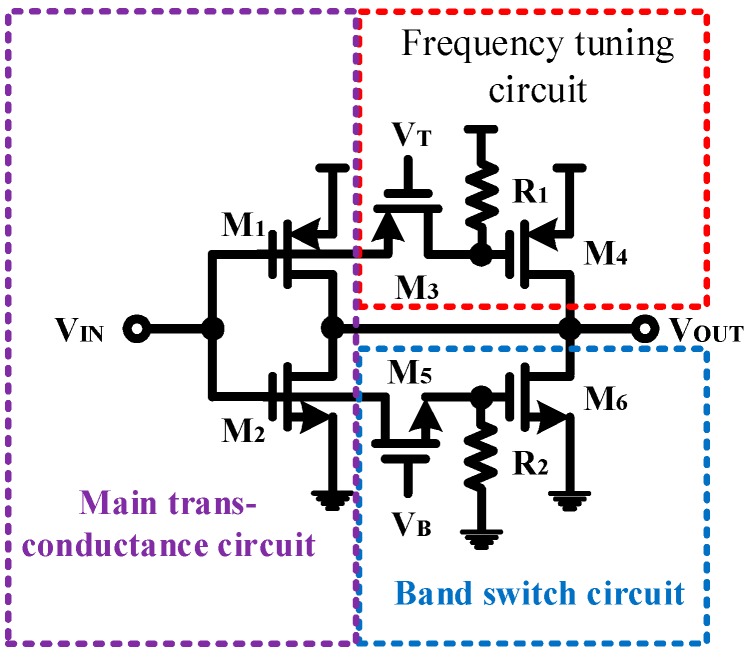
Proposed single-ended delay cell.

This work presents a dual-band six-phase voltage-control oscillator that is based on the proposed single-ended delay cell. The proposed VCO consists of two three-stage ring VCOs and three phase-shift circuits, which provide six symmetrical phase outputs. Figure 2 and Figure 3 show the proposed VCO and delay cell, respectively. Figure 2 also shows the phase difference between the output signals. Similarly, other even-phase outputs VCO also can be achieved by the proposed architecture and delay cell. For example, two five-stage ring oscillators can obtain 10 symmetrical even-phase outputs, two seven-stage ring oscillators can obtain 14 symmetrical even-phase outputs, and so on.

**Figure 4:**
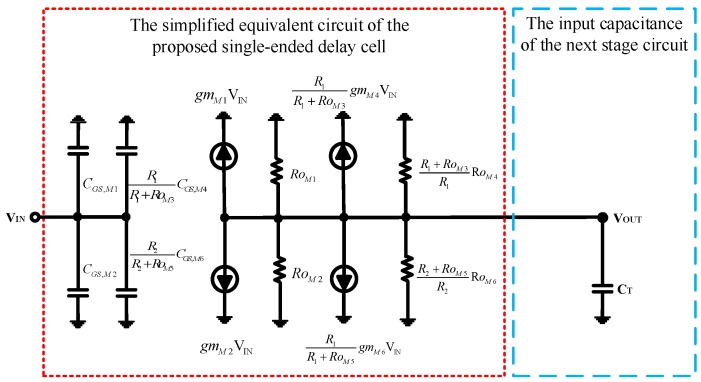
The simplified equivalent circuit of the proposed single-ended delay cell.

The proposed delay cell includes the main trans-conductance circuit, the band switch circuit and the frequency tuning circuit, as shown in Figure 3. Figure 4 shows the simplified equivalent circuit of the proposed single-ended delay cell. The 3 dB bandwidth of the delay cell is determined by the turn on resistance and gate-source parasitic capacitance of the transistor *M*_1_~*M*_6_ (Figure 3), which dominates the output frequency of the VCO, and the 3 dB bandwidth of the proposed delay cell can be approximated as:

(1) $$\omega_{3dB}=\frac{1}{\left(Ro_{M1}∥Ro_{M2}∥\frac{R_{1}+Ro_{M3}}{R_{1}}Ro_{M4}∥\frac{R_{2}+Ro_{M5}}{R_{2}}Ro_{M6})(C_{GS,M1}+C_{GS,M2}+\frac{R_{1}}{R_{1}+Ro_{M3}}C_{GS,M4}+\frac{R_{2}}{R_{2}+Ro_{M5}}C_{GS,M6}\right)}$$

where *Ro_M_*_1_, *Ro_M_*_2_, *Ro*_*M*3_, *Ro_M_*_4_, *Ro_M_*_5_, and *Ro_M_*_6_ are the on-resistances of *M*_1_, *M*_2_, *M*_3_, *M*_4_, *M*_5_ and *M*_6_, respectively; and *C_GS,M_*_1_, *C_GS,M_*_2_, *C_GS,M_*_3_, *C_GS,M_*_4_, *C_GS,M_*_5_, and *C_GS,M_*_6_ are the gate-source parasitic capacitance of *M*_1_, *M*_2_, *M*_3_, *M*_4_, *M*_5_, and *M*_6_, respectively. In order to simplify the equation, the parasitic capacitance of the gate-drain has been ignored. The *C_T_* in Figure 4 is the input equivalent capacitance of the next stage circuit and *C_T_* can be expressed as:

(2) $$C_{T}=C_{GS,M1}+C_{GS,M2}+\frac{R_{1}}{R_{1}+Ro_{M3}}C_{GS,M4}+\frac{R_{2}}{R_{2}+Ro_{M5}}C_{GS,M6}$$

Amplifying the length and width of *M*_4_ by the same scale can greatly increase the on-resistance, *Ro_M_*_4_, and the gate-source capacitance, *C_GS,M_*_4_, of the transistor *M*_4_. For parallel resistors, $\frac{R_{1}+Ro_{M3}}{R_{1}}Ro_{M4}$ can be ignored. For parallel capacitors, $\frac{R_{1}}{R_{1}+Ro_{M3}}C_{GS,M4}$ will be magnified. Reducing the length and width of *M*_6_ by the same ratio can greatly reduce the on-resistance, *Ro_M_*_6_, and the gate-source capacitances *C_GS,M_*_4_ of the transistor *M*_6_. For parallel resistors, $\frac{R_{2}+Ro_{M5}}{R_{2}}Ro_{M6}$ will be amplified. For parallel capacitors, $\frac{R_{2}}{R_{2}+Ro_{M5}}C_{GS,M6}$ can be ignored. For the above reasons, Equation (1) can be simplified as:

(3) $$\omega_{3\mathrm{dB}}=\frac{1}{\left(Ro_{M1}∥Ro_{M2}∥\frac{R_{2}+Ro_{M5}}{R_{2}}Ro_{M6})(C_{GS,M1}+C_{GS,M2}+\frac{R_{1}}{R_{1}+Ro_{M3}}C_{GS,M4}\right)}$$

where *V_B_* in Figure 3 is a band switch signal, which can be used to turn on or turn off the transistor *M*_5_. The transistor *M*_5_, an N-type transistor, is turned off, when *V_B_* = 0. The resistance of *Ro_M_*_5_ is close to ∞. The 3 dB bandwidth of the proposed delay cell can be changed as:

(4) $$\omega_{3\mathrm{dB}}=\frac{1}{\left(Ro_{M1}∥Ro_{M2})(C_{GS,M1}+C_{GS,M2}+\frac{R_{1}}{R_{1}+Ro_{M3}}C_{GS,M4}\right)}$$

In this case, the VCO is operated in low frequency mode. 

On the contrary, when *V_B_* = 1.8, the transistor *M*_5_ is turned off. The resistance of *Ro*_*M*5_ is close to zero. The 3 dB bandwidth of the proposed delay cell can be changed as:

(5) $$\omega_{3\mathrm{dB}}=\frac{1}{\left(Ro_{M1}∥Ro_{M2}∥Ro_{M6})(C_{GS,M1}+C_{GS,M2}+\frac{R_{1}}{R_{1}+Ro_{M3}}C_{GS,M4}\right)}$$

In this case, the VCO is operated in high frequency mode. According to Equations (4) and (5), the proposed VCO can operated in different frequency modes by switching *V_B_*.

For Equation (3), when *V_B_* is fixed, the output frequency of the proposed VCO can also be changed by the resistance *Ro*_*M*3_. For example, the transistor *M*_3_, a P-type transistor is turned on when *V_T_* = 0. The resistance of Ro_M3_ is close to Zero. The 3 dB bandwidth of the proposed delay cell can be changed as:

(6) $$\omega_{3\mathrm{dB}}=\frac{1}{\left(Ro_{M1}∥Ro_{M2}∥\frac{R_{2}+Ro_{M5}}{R_{2}}Ro_{M6})(C_{GS,M1}+C_{GS,M2}+C_{GS,M4}\right)}$$

When *V_T_* increases, the resistance of *Ro*_*M*3_ increases. From Equation (3), when *Ro*_*M*3_ increases, the effect of the gate-source parasitic capacitance *C_GS,M_*_4_ decreases. When *V_T_* = 1.8 V, the resistance of *Ro*_*M*3_ is close to ∞. The 3 dB bandwidth of the proposed delay cell can be changed as:

(7) $$\omega_{3\mathrm{dB}}=\frac{1}{\left(Ro_{M1}∥Ro_{M2}∥\frac{R_{2}+Ro_{M5}}{R_{2}}Ro_{M6})(C_{GS,M1}+C_{GS,M2}\right)}$$

According to the above conclusion, when *V_T_* increases, the 3 dB bandwidth of the proposed delay cell increases. Therefore, *V_T_* can be used to control the 3 dB bandwidth of the proposed delay cell.

Analyzing the three-stage ring oscillator circuit demonstrates that the circuit oscillates only if the frequency-dependent phase shift equals 180° (Barkhausen criterion). Accordingly, each stage contributes a phase shift of 60°, and the oscillation frequency (*ω_osc_*) under this condition is given by:

(8) $$\tan^{-1}\frac{\omega_{osc}}{\omega_{3\mathrm{dB}}}=60^{0}$$

Accordingly,

(9) $$\omega_{osc}=\sqrt{3}\omega_{3\mathrm{dB}}$$

In other words, the output frequency of the VCO can be changed by varying the control voltage (*V_T_*). For example, *M*_3_ is a p-type transistor. According to Equations (3), (6), (7) and (9), the output frequency increases with the control voltage. Therefore, the *Kvco* curve of the proposed VCO circuit has a positive slope. When the system requires that the *Kvco* curve of the VCO has a negative slope, the P-type transistor (*M*_3_) should simply be replaced with an N-type transistor (*M*_3_). In such a case, the output frequency decreases as the control voltage increases and so the *Kvco* curve of proposed VCO circuit has a negative slope. Therefore, whether the transistor (*M*_3_) is NMOS or PMOS determines whether the linear frequency-voltage relationship of the proposed VCO is positive or negative. This advantage also meets the diverse needs of sensor systems.

When the output frequency is changed with the input voltage (*V_T_*), no DC current flows through the transistor *M*_3_. Therefore, the DC operating point and power consumption of the circuit will not change much, reducing the design complexity of the sensor circuit system.

**Figure 5:**
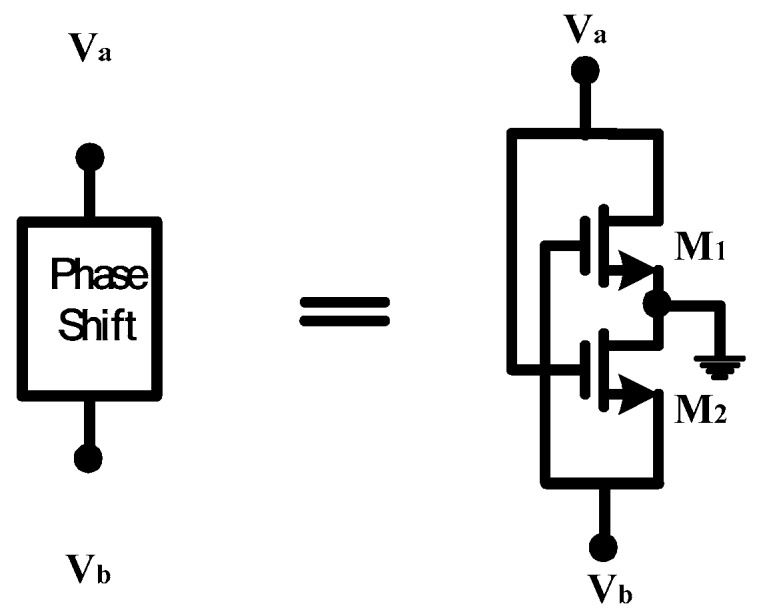
Proposed phase shift circuit.

Figure 5 shows the phase shift circuit in Figure 2. The phase shift circuit is a cross-coupled pair circuit, which has two nodes whose phase difference can be adjusted to 180 degrees. The three-stage ring VCOs provide a phase difference of 120 degrees between the stages. For the above two reasons, the proposed VCO, shown in Figure 2, can provide six symmetrical phase outputs.

**Figure 6:**
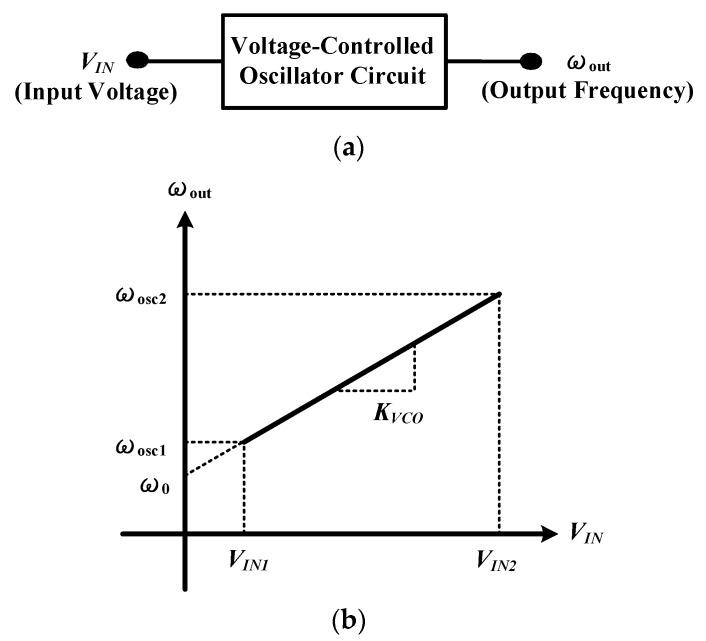
(**a**) Input and output relationship of VCO, (**b**) the slope of the input voltage to the output frequency.

The output frequency of an ideal voltage-controlled oscillator is a linear function of its input voltage (shown in Figure 6a), and can be expressed as:

(10) $$\omega_{osc}=\omega_{0}+Kvco\times V_{IN}$$

where *ω*_0_ represents the intercept corresponding to *V_IN_* = 0 and *Kvco* denotes the “gain” or “sensitivity” of the circuit [15]. In other words, the slope of the input voltage to the output frequency is defined as *Kvco*, which can be obtained by inputting different voltages and their corresponding output frequencies (shown in Figure 6b).

For a given noise amplitude, the noise in the output frequency is proportional to the VCO gain (*Kvco*) [22]. Therefore, based on the same operating bandwidth condition, the band control circuit can effectively reduce the gain of VCO (*Kvco*), which results in improved VCO jitter performance. The proposed VCO circuit can be operated in high- or low-frequency mode by turning on (*V_B_* = 1.8) or off (*V_B_* = 0) transistor *M*_5_, which thus acts as a band switch for the VCO. Through this band switching function, the jitter performance of proposed VCO is improved.

The proposed single-ended delay cell with the current reuse technique provides a higher trans-conductance (gm) than conventional differential delay cell topologies without a higher power dissipation. Therefore, the recommended VCO is more energy-efficient under the same performance conditions. 

## 3. Measurement and Simulation Results

**Figure 7:**
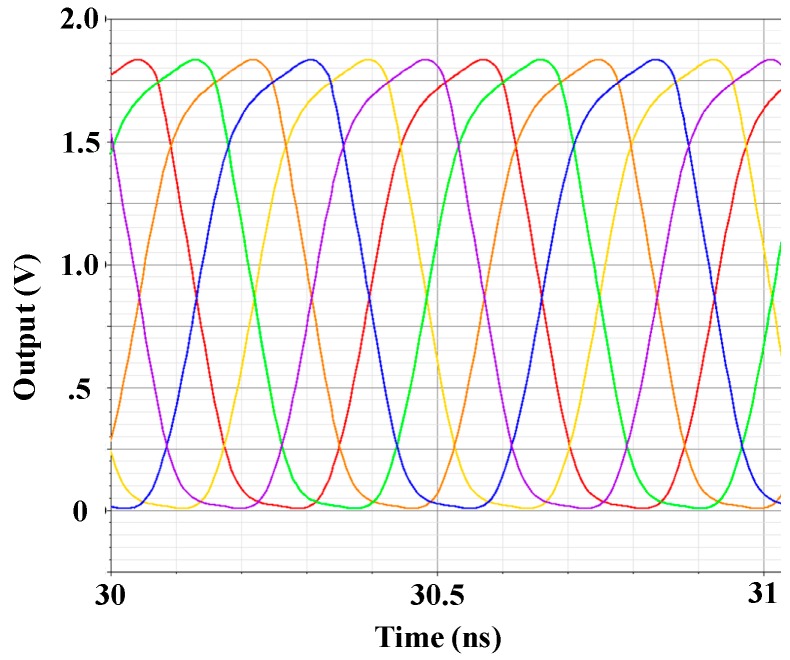
The transient simulation waveforms of the proposed six-phase output VCO.

**Figure 8:**
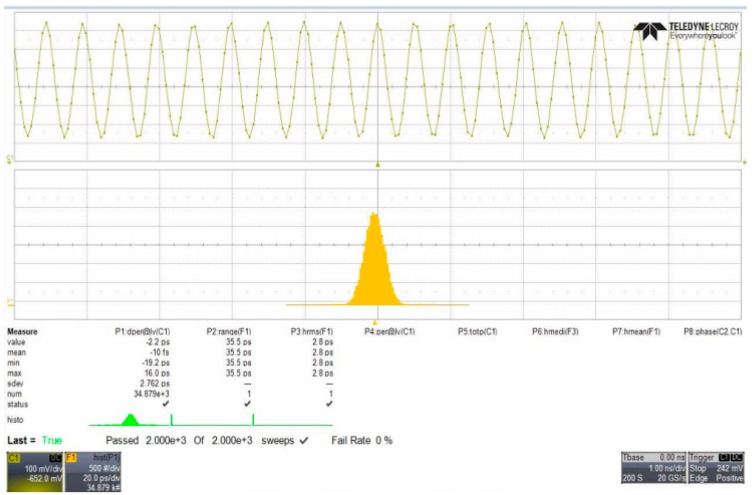
Peak-to-peak jitter and root mean square jitter of proposed VCO.

**Figure 9:**
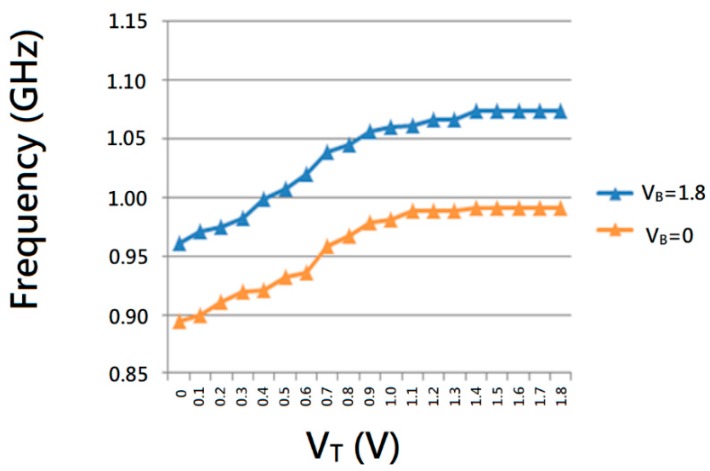
*Kvco* curve of proposed VCO at different frequency mode.

The proposed VCO is fabricated using a United Microelectronics Corporation 0.18 μm RF CMOS process with 1.8 V supply voltage. Figure 7 shows the transient simulation waveforms of the proposed six-phase output VCO, which shows that the proposed VCO has six symmetrical output phases. Figure 8 plots the peak-to-peak jitter and root mean square jitter of the proposed VCO, which are 35.5 ps and 2.8 ps, respectively (at an operating frequency of 1 GHz), showing that the proposed VCO exhibits favorable jitter performance. Figure 9 reveals that the proposed VCO can be operated in different frequency modes using the proposed band switching technique. In Figure 3, the band switch control voltage is 1.8 V (*V_B_* = 1.8); therefore, the proposed VCO can be operated in high-frequency mode. By contrast, when the band switch control voltage is 0 V (*V_B_* = 0), the proposed VCO can be operated in low-frequency mode. The proposed VCO can thus operate in a tunable frequency mode at similar power consumptions and can cover the 890–1080 MHz band.

**Figure 10:**
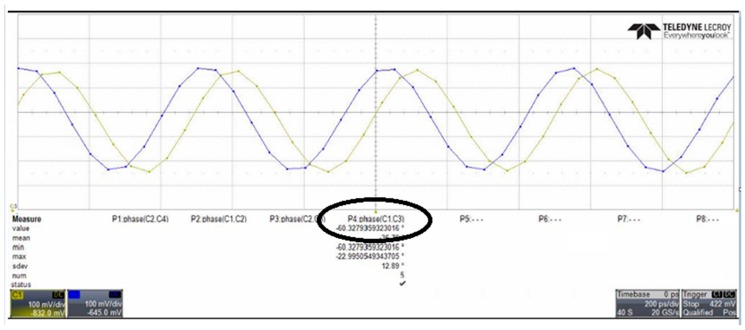
A 60.32° phase shift occurs between the two output signals.

**Figure 11:**
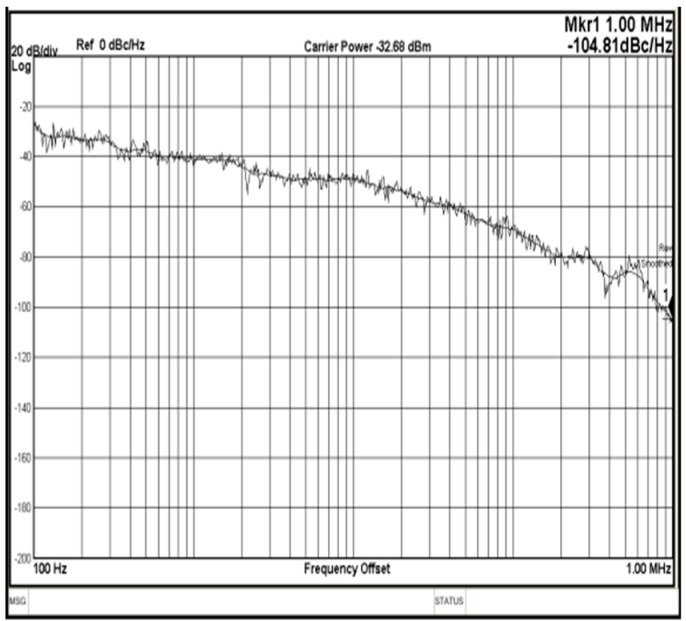
Measured phase noise curve of the proposed VCO (at oscillation frequency of 1 GHz).

**Figure 12:**
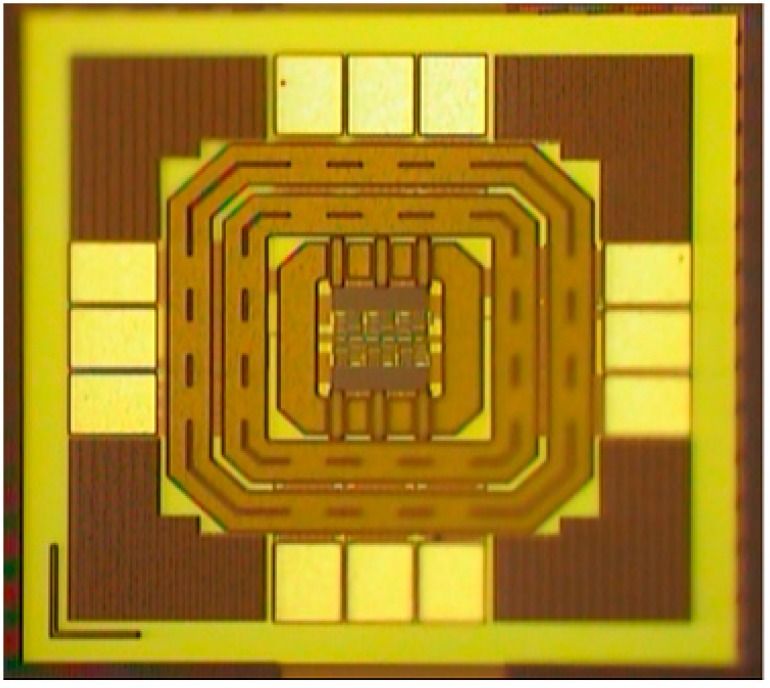
Microphotograph of the proposed VCO.

Figure 10 shows an approximately 60.32° phase difference between the two output signals, meaning that each stage of the proposed VCO contributes a frequency-dependent phase shift of approximately 60.32°. Since the proposed VCO is implemented with six identical delay cells and three identical phase shift circuits, the phase shifts in all delay cell stages are similar. Therefore, no phase mismatch occurs in pre-simulation states. However, a 0.32-degrees mismatch has been discovered in measurement result. It is because the phase error is caused by the parasitic elements mismatch in layout states. Therefore, the phase mismatch in the proposed VCO can be improved by using better layout techniques. Comparing Figure 7 and Figure 10, the output frequency and voltage swing of the measurement result are smaller than the transient waveform. That is caused by the parasitic capacitance of the wire during circuit layout. This can be improved by improving the layout skills. Figure 11 reveals how the phase noise is calculated using the Agilent phase noise measurement solution. The phase noise in the proposed VCO is lower than −104.8 dBc/Hz (at 1 MHz offset). Figure 12 shows a chip micrograph of the proposed VCO. The maximal power consumption is approximately 6.4 mW at a supply voltage of 1.8 V. The core area of the proposed four-phase VCO is 0.195 × 0.208 mm^2^ (core only).

Table 1 presents the characteristics of the proposed and other VCOs. Compared with [8], the proposed VCOs are based on single-ended delay cells, but the proposed VCO has a higher operating frequency, a lower jitter, and more output phases than the VCO in [8]. The VCO in [9] is based on a differential delay cell. The proposed VCO requires less than half of the power consumption in [9] at the same output phase condition and close operating frequency. [10] is also based on a differential delay cell, which has a higher operating frequency, but its performance is not good in power consumption, phase noise, and power supply voltage. Moreover, the proposed VCO and the VCO of [8] have band switching function, which can effectively improve the jitter performance of VCO. The proposed VCO exhibits less jitter and consumes less power than the other VCOs. Furthermore, it has symmetrical six output phases and is suitable for use in sensor systems.

## 4. Conclusions

The data collection for the Internet of Things and big data requires many different sensor devices. However, these sensor devices require a VCO to generate a reference clock frequency or local oscillator frequency. Therefore, improving the performance of the sensor device through the improvement of the VCO is an important point of research. This paper proposes a novel VCO and a novel single-ended delay cell. The proposed VCO can have the advantage of the single-ended delay cell and the differential delay cell. Hence, the proposed VCO has the advantages of large output swing, small chip area, low dissipated current, simple design, and good jitter performance. The proposed VCO also has odd-phase and even-phase output functions. Moreover, the proposed VCO can convert between a positive slope and a negative slope by replacing a transistor. For the above reasons, the proposed VCO has advantages in terms of performance or design flexibility. Therefore, the proposed VCO meets the requirements of various sensor systems. 

## Figures and Tables

**Table 1:** A comparison of the proposed VCO to other existing VCOs.

	This Work (Meas.)	[8] (Meas.)	[9] (Meas.)	[10] (Meas.)
Operation Frequency	890~1080 MHz	12.6~48 MHz	1.77~1.92 GHz	2450 MHz
Supply Voltage	1.8 V	1.2 V	1.8 V	2.5 V
Peak-to-peak jitter	35.5 ps (at 1 GHz)	525 ps (at 25 MHz)	NA	NA
RMS jitter	2.8 ps (at 1 GHz)	78.2 ps (at 25 MHz)	NA	NA
Phase noise	−104	−109	−123 dB	−96
	(phase noise at 1 MHz offset)	(phase noise at 1 MHz offset)	(phase noise at 10 MHz offset)	(phase noise at 1 MHz offset)
Process	0.18 μm	0.18 μm	0.18 μm	0.28 μm
Architecture	Single Ended	Single Ended	Differential	Differential
	(6 stage)	(4 stage)	(3 stage)	(2 stage)
Output phase	6	4	6	4
Power consumption (mW)	6.4	1.2	13	19.2
Band switch	Yes	Yes	No	No
